# Ethnic stereotypes and the underachievement of UK medical students from ethnic minorities: qualitative study

**DOI:** 10.1136/bmj.a1220

**Published:** 2008-08-18

**Authors:** Katherine Woolf, Judith Cave, Trisha Greenhalgh, Jane Dacre

**Affiliations:** 1 Academic Centre for Medical Education, University College London Division of Medical Education, London N19 5LW; 2 Open Learning Unit, Department of Primary Care and Population Sciences, University College London

## Abstract

**Objective** To explore ethnic stereotypes of UK medical students in the context of academic underachievement of medical students from ethnic minorities.

**Design** Qualitative study using semistructured one to one interviews and focus groups.

**Setting** A London medical school.

**Participants** 27 year 3 medical students and 25 clinical teachers, purposively sampled for ethnicity and sex.

**Methods** Data were analysed using the theory of stereotype threat (a psychological phenomenon thought to negatively affect the performance of people from ethnic minorities in educational contexts) and the constant comparative method.

**Results** Participants believed the student-teacher relationship was vital for clinical learning. Teachers had strong perceptions about “good” clinical students (interactive, keen, respectful), and some described being aggressive towards students whom they perceived as quiet, unmotivated, and unwilling. Students had equally strong perceptions about “good” clinical teachers (encouraging, interested, interactive, non-aggressive). Students and teachers had concordant and well developed perceptions of the “typical” Asian clinical medical student who was considered over-reliant on books, poor at communicating with patients, too quiet during clinical teaching sessions, and unmotivated owing to being pushed into studying medicine by ambitious parents. Stereotypes of the “typical” white student were less well developed: autonomous, confident, and outgoing team player. Direct discrimination was not reported.

**Conclusions** Asian clinical medical students may be more likely than white students to be perceived stereotypically and negatively, which may reduce their learning by jeopardising their relationships with teachers. The existence of a negative stereotype about their group also raises the possibility that underperformance of medical students from ethnic minorities may be partly due to stereotype threat. It is recommended that clinical teachers be given opportunities and training to encourage them to get to know their students as individuals and thus foster positive educational relationships with them.

## Introduction

Medical students from ethnic minority backgrounds make up about 30% of the medical student population in the United Kingdom^1^; however, UK medical students and doctors from ethnic minorities significantly underperform in assessments compared with their white counterparts.^2^ ^3^ ^4^ ^5^ ^6^ ^7^ ^8^ Similar findings have been reported in the United States^9^ and Australia.^10^ Some evidence suggests that the ethnic gap might be greatest in assessments of clinical knowledge and skills, but it is also present in machine marked tests of basic medical knowledge.^11^ ^12^ Students from ethnic minorities enter medical school with slightly lower school leaving examination grades than white students, but this only partly explains the ethnic gap seen later.^1^

In the United States, academic underperformance of people from ethnic minorities, particularly African-Americans, has been explained by the theory of “stereotype threat,” according to which members of negatively stereotyped groups can feel sufficient anxiety at the prospect of being negatively stereotyped that they underperform in test situations.^13^ ^14^ Might stereotype threat explain the underachievement of UK medical students from ethnic minorities? The primary problem in applying this theory to UK medical students is that although there is a pervasive negative stereotype that African-Americans are less intelligent than European Americans, it is not clear where there are negative stereotypes about UK medical students from ethnic minorities.

We sought to explore stereotype threat and other factors that might affect students from ethnic minorities learning in clinical environments, in a sample of year 3 (first year clinical) medical students and a sample of their clinical teachers. We aimed to explore any stereotypes about medical students from ethnic minorities and to generate hypotheses to explain underachievement in year 3 medical students from ethnic minorities.

## Methods

We gathered data from year 3 medical students and their clinical teachers using one to one, face to face interviews initially. After 12 interviews it became clear that students did not feel comfortable discussing ethnicity and therefore we used focus groups of single ethnicity to collect the rest of the data on students.

### Sampling strategy and recruitment

#### Clinical teacher one to one interviews

Clinical teachers were purposively selected from a sampling frame including senior surgeons, doctors, general practitioners, and tutors in clinical skills. Data from one London hospital’s website showed that in 2003 69% of consultants were men, 88% were white, 1% were black, and 9% were Asian (www.royalfree.nhs.uk/doc/240604/AppendixF.doc). The sampling frame was designed to reflect these demographics. Forty clinical teachers were sent a letter from JD (vice dean) inviting them to take part in a 20 minute semistructured interview. During the sampling phase participants were assigned a sex and ethnic group on the basis of their names, and they were subsequently asked to self report ethnicity using the 2001 UK census categories (www.statistics.gov.uk/census2001/profiles/commentaries/ethnicity.asp#background). Non-responders received up to three email and telephone reminders.

#### Student one to one interviews

Students were purposively sampled to provide “information-rich cases”^15^ using the demographics of the year 3 student population as a sampling frame (information on self reported sex and ethnicity was obtained from student records). Forty nine students were emailed to ask whether they would spend about 30 minutes being interviewed about their experiences of clinical teaching for a PhD project about the factors that affect performance at medical school.

#### Student focus groups

Of the 360 year 3 medical students of known self reported ethnicity (94.5% of the total year group), students from the three largest ethnic groups—white, Indian, and Pakistani—were invited to participate. To increase homogeneity within groups and therefore increase the chances of participants feeling comfortable and able to talk freely,^16^ we organised potential participants by ethnicity and year 3 clinical firm grades into six groups: Indian high achieving, Indian low achieving, Pakistani/Bangladeshi high achieving, Pakistani/Bangladeshi low achieving, white high achieving, and white low achieving. We also invited students who had previously expressed an interest in taking part in the one to one interviews. We made no attempt to exclude friends or acquaintances, with the aim that some collective remembering of events might be captured^17^ and also that participants would be more likely to raise sensitive topics if accompanied by friends.

We sent students a maximum of three emails and one text message reminder. If students declined, they were sent another email asking their reasons.

### Procedure

#### One to one interviews: students and clinical teachers

KW (British white female junior faculty member) carried out the interviews in 2005 and 2006 at a time and in a private environment convenient for participants. Throughout the participants were encouraged to expand and clarify their answers. Interviews ranged from five minutes to 1.5 hours (average 15 minutes).

#### Focus groups: students

KW moderated while SB (British black Caribbean female psychology undergraduate student) took notes. KW explained the purpose of the group. Throughout the session storytelling was encouraged and participants were prompted to expand on and explain their comments and to comment on others’ contributions. Wherever appropriate KW remained silent to allow conversations between participants. At the end of the session the students were given a stamped addressed envelope and asked to contact KW by post, email, or telephone if they thought of anything else they wanted to say.^16^

### Questions

We used open questions to provide opportunity for discussion. Questions concerned participants’ experiences of teaching (clinical teachers) and learning (students) in the clinical environment; differences between students that might make some learn a lot and others not as much in the same situations. The focus groups also included an open question about what it meant to be from a particular ethnic group (see web extra for questions). One of the aims of the study was to generate hypotheses to explain underperformance in students from ethnic minorities and therefore participants were specifically asked what they thought of research findings that had shown that such medical students underperform academically compared with white students.

### Analysis

KW transcribed the data verbatim. KW and JC read and reread and familiarised themselves with the transcripts. We analysed all data using stereotype threat as a theoretical framework. KW searched the data for the ways in which participants portrayed white students and those from ethnic minorities in clinical teaching contexts. Similarities and differences between the descriptions used by clinical teachers and students were sought. KW and JC discussed the stereotypes that emerged from the data and, through discussion, coded them for how they related to different aspects of teaching and learning in the clinical environment. Throughout the analysis the interpretation was compared with the verbatim data. Opposite examples were sought and used to refine the analysis.

Participants gave oral informed consent at the start of the interviews or focus groups. One clinical teacher declined to be audio-recorded, but agreed to notes being taken and used for research purposes.

## Results

Overall, 26 of 40 clinical teachers agreed to be interviewed (65%), 12 did not answer, and two declined: one was too busy and one did not give a reason (table 1). Of the 49 students invited to participate, 21 agreed to be interviewed (43%). Twelve interviews took place—six with white students and six with students from ethnic minorities. Five of the students were men and seven were women. High achievers were more likely to attend the focus groups than low achievers (19.3% *v* 9.0% of those invited). The Pakistani/Bangladeshi lower achieving group had to be abandoned as a result of non-attendance. If necessary, students were interviewed in pairs (table 2).

**Table 1 tbl1:** Number of clinical teachers interviewed by specialty, sex, ethnic group, and year of qualification

Specialty	Men	Women	White	Ethnic minority	Total	Median year (range) of qualification
Consultant	10	2	9	3	12	1983 (1965-87)
Consultant surgeon	5	1	5	1	6	1987 (1977-90)
General practitioner	0	5	4	1	5	1990 (1984-98)
Nurse (clinical skills tutor)	0	2	2	0	2	NA*
Total	15	10	20	5	25	1985 (1965-98)

*Year of qualification not given for clinical skills teachers to ensure anonymity.

**Table 2 tbl2:** Focus group invitees and attendees by ethnic group and sex

Group	No in year 3	No invited	No (%) attended	No of male attendees	No of female attendees
Indian group:					
High achievers	30	15	3 (20)	2	1
Low achievers	43	14	2 (14)	0	2
Total (% of total year)	73 (20)	29	5 (17)	2	3
Pakistani/Bangladeshi group:					
High achievers	13	13	2 (15)	0	2
Low achievers	11	11	0 (0)	0	0
Total (% of total year)	24 (7)	24	2 (8)	0	2
White group:					
High achievers	92	26	6 (23)	1	5
Low achievers	41	16	2 (13)	2	0
Total (% of total year)	133 (37)	42	8 (19)	3	5

### Importance of student-teacher relationship to learning

The student-teacher relationship was described as one of the most important factors in determining the quality of learning. Teachers believed that to foster these relationships it was important to find out about students’ individual educational and pastoral needs and make efforts to tailor the teaching to those needs. They enjoyed and put most effort into teaching students who interacted with them, asked questions, and seemed otherwise keen and enthusiastic (for example, by having done some preparatory reading) whereas quiet students were perceived as unresponsive, unenthusiastic, and unappreciative. Although most teachers realised that students generally did not like being humiliated, ignored, or otherwise disrespected, some teachers (mainly white men) described how they could behave antagonistically towards students they thought were not making sufficient effort to learn (box 1).

**Box 1 Antagonistic behaviour towards students**

The more interest you show in them [students] and the more individual it is as an experience, I think the more responsible they are about letting you know what is happening and feeling that they have, it’s in their interest to turn up (teacher 6: female consultant, white)
KW: so what’s it like when the students are quiet?
It’s actually quite difficult. Um, you think “oh God is there something wrong with the teaching?” (teacher 22: female general practitioner, Asian)
We’re busy, we’re fairly bullish […] If you give me five keen students, they get a fantastic deal. If you give me five quiet reticent students they get a crap deal (teacher 25: male surgeon, other ethnic group)
A little bit of fear ain’t a bad thing from where I come from. I may push someone over the edge and they’ll probably commit suicide and I’ll be terribly sorry but that’s a risk I will take […] If they’re prepared to work together, I will work with them, literally the whole time on the firm if they’re not, don’t bother me about it, go and get a life because you’re not going to enjoy it (teacher 18: male surgeon, white)

Students described how they appreciated the teachers’ efforts and learnt most when teachers interacted with them. They did not like being taught by teachers who seemed unenthusiastic, or who ignored or humiliated them. They explained that they would not feel able to interact or might not attend those sessions (box 2).

**Box 2 Student-teacher interactions**

I think most of when I learn when there’s lots of perhaps student-teacher interaction (student 2: male, Asian)
[The teaching session] was really like one-on-one, you got loads of attention. And you didn’t have the chance to switch off, you know um, they also seemed really keen to make sure that we knew stuff. Like, they actually cared whether we did or we didn’t […] [The teacher] was like “so, where did you grow up? Where’s your family home?” Like, “why did you decide to do medicine?” […] “So what do you do in your free time?” […] And he wrote it all down (student 11: female, white)
[Bullying tactics] will tend to make [students] quieter, they don’t tend to ask questions and they tend to leave sessions confused and I think that leads to them thinking “there’s no point in me learning” (student 1: female, Asian)

### Perceptions about “typical” Asian medical students

As well as having perceptions about “good” clinical students, clinical teachers had perceptions about Asian medical students. Students, including Asians, had similar perceptions. We present the way Asian medical students were described in terms of their relationship with books, with patients, and with teachers.

In terms of their relationship with books, the “typical” Asian student was viewed as conscientious, hard working, and bright. Non-Asian participants were more likely to qualify this picture with a flip side: that being over-reliant on books made the “typical” Asian student inflexible and less able to adapt to new ways of behaving. Thus it was perceived that although Asian students came to medical school with excellent examination results, learning the nebulous art of clinical medicine required flexible learning from novel situations, and that such students might struggle in these circumstances (box 3).

**Box 3 Perceptions about the “typical” Asian medical student’s relationship with books**

Students that are of South Asian or Indian origin, tend to be, or come across as being far more academically knowledgeable and they can justify what they’re doing and they’re very very bright, but actually putting that into practice and both with communication and practical skills doesn’t seem to gel that well (teacher 11: female clinical skills tutor, white)
The Asian students they are very keen, very studious, industrious (teacher 24: male consultant Asian other)
Student N, female, and student M, male (white high achieving group):
  N: the Asian students I know, know a lot from books, a lot of the technical detail, whereas I don’t know any of that stuff, that’s just not how I learn
  M: yeah, so in the MCQs and EMQs [written assessments] and stuff which are knowledge based I wouldn’t be surprised if the [ethnic] differences [in attainment] aren’t there
I think, also with ethnic minorities in general they tend to study a lot. Which is fine, that’s just the way the culture is, it’s more, especially if I’m going [laughs] especially in Asian cultures […] And so when it comes to clinical setting they find it difficult (student 2: male, Asian)

From personal experience one Indian student from the high achieving group believed that Asian students may have worked particularly hard at school, and would now do the minimum to pass medical school exams:

“Instead of like, wanting to be the best I can be in the year [like I did at school], I’m more like ‘well as long as I pass, that’s all right’ […] And maybe that’s like the attitude of … I dunno I’m saying, I’m guessing, of a few, fair proportion of the non-whites” (student K: male, Indian high achieving group)

In terms of their relationships with patients, non-Asian participants perceived the “typical” Asian student as a poor communicator, either because of varying degrees of linguistic problems (for example, accent) which (allegedly) made them feel under-confident, or because they were culturally more formal than white students (box 4).

**Box 4 Perceptions about the “typical” Asian medical student’s relationships with patients**

It’s much more common to come across an Asian student, even if they’re English-born, that has formal relationships with patients than it is to find a, a white British-born person having formal relationships with patients (teacher 10: female general practitioner, white)
I’ve had people [students] who are, for example, from the Far East who are extremely polite you know very polite and so on, but may come across um, in not quite the same, just because they’re you know of their culture being extremely polite, may not come across [to patients] as well (teacher 3: male consultant, Black African)
You get a lot of Asians or some whites that are not maybe as good at speaking to the patients. I know this is a real stereotype (student 10: female, other ethnic group)

In terms of their relationships with teachers, the “typical” Asian student was perceived by non-Asian teachers and one non-Asian student as shy, quiet, reserved, and under-confident (box 5). Clinical teachers thought this was because Asians were overly respectful of authority.

**Box 5 Perceptions about the “typical” Asian medical student’s relationships with teachers**

When you look back at those failures and those failures of um, being, um, you know, that ethnic minority failing group, those I believe are chronically under-taught, they’re chronically ignored, chronically under-taught, chronically ignored, and they set it up (teacher 15: female general practitioner, white)
Some of these sweet little Asian girlies are very hard to get through to. I’m quite a physically biggish sort of chap, maybe that’s another factor. I’m older, obviously that’s a factor. I’m male. I’m … they don’t communicate terribly well (teacher 2: male consultant, white)
You do get a lot of quiet Asian girls in particular (student 10: female, other ethnic group)

These attributes were perceived as partly the result of pressure from family to conform to Asian cultural norms, which required young Asians to work hard, have professional jobs, respect their teachers, and get married at an appropriate time to an appropriate person (box 6). The “typical” Asian student was seen by Asian and non-Asian participants as more likely to be studying medicine to conform to their parents’ wishes; and non-Asians were more likely to equate this with also having led a sheltered life and being less mature and autonomous. Differences in motivation (“to please parents” rather than internal motivation) were seen by participants from different ethnic groups as indicating that Asian students would be less likely to be deep learners, caring communicators, active participants in their learning, and ultimately good doctors.

**Box 6 Perceived motivational factors**

You sometimes find that students who are incredibly disillusioned say “I went in to medicine because of this, that and the other, because my parents wanted me to” […] My parents certainly wanted me to become a doctor but I wanted to. Um, it worked that way […] I’m speaking from an Asian background not for anyone else. Um, it’s quite well known (teacher 22: female general practitioner, Asian)
The Asian, first degree coming over from Kenya or wherever, they came over in the sixties when Idi Amin kicked them out, they’re very keen on their children achieving excellent attributes. So their children bloody well have to work, there’s a work ethic at home, um and they get three A grades at A level so the authorities let them in because they think three As at A level’s a good thing, which I think is bonkers […] I don’t think your ability at physics A level is in any way related to your ability to be a good doctor (teacher 2: male consultant, white)
There’s a stigma of sort of ethnic families wanting their children to do best and then there’s the whole doctor, lawyer, you know, get the upper, upper rank jobs or whatever they’re called and so I suppose if they’re thinking “oh bollocks, I’ve got to choose between three jobs, I’ll choose the doctor then” (student 12: male, white)
You do get a lot of Asian families who push their children to be doctors […] Even if we’re born and bred here and we’ve lived here for about 30 years, it’s still the kind of thing: if your son’s a doctor that’s fine (student 2: male, Asian)

Direct discrimination in teaching or assessments could in theory explain academic underperformance by students from ethnic minorities. There was no evidence from the data to suggest that students perceived themselves as victims of racism; indeed some specifically said they were not.

### Perceptions about “typical” white students

The idea of the “typical” white student was less well developed; however, white students were perceived by teachers and students as being autonomous learners who were dedicated and self motivated; were tough but sociable team players, as evidenced by their love of the physical team sport rugby; and were confident, outspoken, and good communicators (especially the women). Perceptions of white students were not entirely positive: sometimes they were described as pushy or arrogant, although these characteristics were deemed likely to help students succeed (box 7).

**Box 7 Perceptions about the “typical” white student**

We’ve set up a medical school and higher education which is largely based on the values of a bunch of middle class white people who talk to their children and expect their children to be out there and up there […] And will trade a little bit of politeness for a bit of independence and creativity (teacher 15: female general practitioner, white)
White female students seem to have, for me, the best communication skills with patients. And be most patient-oriented in their approach (teacher 11: female clinical skills tutor, white)
Because I play sports and other such things that I’ve naturally always had like, like even now I’ve got a few white mates and stuff like that (student K: male, Indian high achieving group)
The white people who get into medical school, they’re just across the board they’re more motivated and are doing it for the right reasons and they always have that in mind (student B: female, Indian high achieving group)

## Discussion

Teachers of clinical medical students, and the students themselves, have strong perceptions about “typical” Asian students, and there is a systematic mismatch between these perceptions and the (equally strong) perception of what makes a “good” clinical student. These findings are consistent with the hypothesis that negative stereotypes of Asian medical students exist. This may have numerous implications for teaching and learning.

### Strengths and weaknesses of the study

This qualitative work has provided a greater understanding of the possible reasons for the academic underperformance of medical students from ethnic minorities—an important and under-explored area. The strong theoretical underpinnings of the data analysis were useful in organising the data meaningfully and in generating hypotheses for future testing on the ways in which stereotyping, teacher-student interactions, and performance are related. Both students and teachers were interviewed, which provided triangulation of the results, and the data were analysed by two researchers with different backgrounds—one non-clinical psychology researcher and one clinical teacher and medical educationalist, which improved validity and reliability.

Our study has some limitations. The study design was based on what students and teachers said they thought and did, not on direct observation of what they actually did. The triangulation of results suggested these descriptions were valid—for example, some teachers said they made the effort to get to know their students as individuals to establish a relationship, and some students said they appreciated it and thought they learnt more when teachers made the effort to get to know more about them.

The sex, ethnicity, and age of the interviewer (British female, white) may have affected participants’ discussion of certain topics. For example, KW perceived that white clinical teachers who had positive views about white female medical students and rather more negative views about Asian or male medical students felt comfortable discussing these views with her in a way that they may not have had she been an Asian medical student. That students in the one to one interviews felt uncomfortable discussing ethnicity means that potentially important topics may not have been covered. This taboo has been found previously with medical students^18^ and was addressed by running the focus groups, which seemed to have the desired effect of encouraging students to talk more freely about ethnicity while not compromising overall validity: students in the one to one interviews and focus groups gave similar answers to non-controversial questions, but additional themes arose in the focus groups.

The low participation rate in the focus groups and the relatively low numbers of non-white clinical teachers may have introduced a systematic bias. This is important as low achieving students were less likely to attend the focus group than high achieving students, and teachers from ethnic minorities may have had different ideas from white teachers. This was an exploratory study designed to open up areas for further research: one of the key findings was the difficulty in engaging participation from particular groups of students. This may merit further investigation.

Generalisability is a problem inherent in all qualitative studies. For example, the terms “ethnic minority,” “non-white,” and “Asian” are not considered interchangeable in many contexts; however, in the context of a medical school where most of the students from ethnic minorities are of South Asian origin (Indian, Pakistani, Sri Lankan), when participants spoke of “ethnic minorities” or “non-whites” these terms were interpreted as meaning “Asian”—a term used by many of the Indian and Sri Lankan participants to refer to themselves. The aim of this study, however, was not to produce an estimate of the extent or impact of stereotype threat on a particular population or to test hypotheses about it, but to provide a preliminary exploration of the topic with a view to prompting reflection by teachers, students, and policy makers, and informing future research.

### Strengths and weaknesses in relation to other studies

Much of the previous research into ethnicity in medical education has related to measuring differences in performance between ethnic groups.^5^ ^6^ ^11^ Although these differences are important, these studies provide little insight into the reasons why this gap exists. A few previous qualitative studies have explored issues surrounding ethnicity in undergraduate medical education. Sinclair^19^ describes medical students from University College London in the 1990s using racial stereotypes, although he particularly comments on stereotypes of black students. In our study most comments about black students concerned their absence from the medical student population. In a more recent study, London medical students talked about how “quiet Asian girls” were “less able” and treated less well by consultants,^20^ which is similar to the findings of this study. A study on “everyday racism” at a Canadian medical school described ethnic stereotypes of medical students, but this time in the context of white students being the stereotypical medical student and therefore the type of person that patients expected to see.^21^ The data in the above studies were not specifically analysed in relation to the effects of ethnic stereotyping on learning, and therefore our study adds a new dimension to previous findings. Previous studies have also found that students from ethnic minorities can experience marginalisation and segregation.^18^ ^21^ The relationships between students and how these affect learning are no doubt extremely important. Our study did look at the ways in which students from different ethnic groups perceive each other but not the ways in which they thought they behaved towards each other.

### Meaning of the study

One study^14^ argued that for stereotype threat to have an effect students have to be concerned that people will make negative assumptions about them on the basis of the stereotype and that by behaving in a particular way they are conforming to that negative stereotype. According to that study this concern can make students sufficiently anxious to negatively affect their performance, or can prompt them to try and behave in a way that counters the stereotype. One Asian student in this study described precisely this phenomenon: she recalled hearing clinical teachers talking about the number of students from ethnic minorities at medical school and how she believed that teachers presumed that as an Asian student with medical parents she had been forced into medicine (the stereotype). She perceived they purposely made life harder for her, resulting in her feeling under psychological pressure (stereotype threat) and forced to prove that she was worthy of being at medical school. To prevent people making stereotypical assumptions about her she avoided telling people about herself. In another example, a male Asian student described how he thought he needed to work extra hard to combat any possibility that he would be negatively stereotyped as an underachiever. A female Indian Muslim student said that she believed that her seniors assumed she was “substandard” on the basis that she wore a headscarf. It is important to note that those last two students did not describe feeling that way because of overt racial discrimination from clinical teachers, yet they thought that negative stereotypes about their group could adversely affect them in a clinical setting. These are just three examples of the different ways in which the feeling of being stereotyped may lead students from ethnic minorities to feel and behave in a particular way; however, the relation between stereotyping and performance is not simplistic or deterministic and is probably moderated by several different student dependent factors, such as psychological resilience and personality. Further research is required to determine the relationships between those factors.

Research shows that relying on stereotypes can stop people from searching out information that conflicts with the stereotype (so called confirmation bias^22^). One study^14^ recommends that to combat the effects of stereotyping, teachers should get to know their students individually. Even in the absence of stereotype threat, however, Asian students who are stereotyped and therefore not seen as individuals are less likely to have effective educational relationships with their teachers.^23^ ^24^ Students who perceive that they are being stereotyped may find it more difficult to learn, as negative emotions interfere with learning.^23^ Clinical teachers with stereotypical views of Asian students may feel less positive about teaching them. This may lead to a negative spiral whereby those students come to feel less motivated to learn, thus making them quieter and less interactive, which then leads clinical teachers to be more negative towards them, and so on.

On the basis of the findings presented here we recommend that clinical teachers should make efforts to get to know their students as individuals, and that employers should provide the training and infrastructure to help them achieve this task. This will benefit most students but has the additional advantage for students from ethnic minorities of countering the effects of stereotyping, including stereotype threat. The following quote from a clinical teacher vividly illustrates how getting to know students, even briefly, can enable stereotypical views to be overcome and constructive relationships to be fostered, leading to positive learning experiences for students:

“Before [the patient entered] we [the student and I] talked briefly […] just a brief chat about “who you are, where you come from, um, where you’re up to, what are your interests, what have you just done?” […] Suddenly my interest in her shifted and my perception of her changed and my respect for her went up and that’s awful, but I didn’t just see ‘a student’ another student, another Indian, you know, young Asian, frighteningly thin student in front of me, I actually saw this person who was actually quite interesting […] When patients came in, I don’t know, it was just easy to engage her” (teacher 15: female general practitioner, white)

### Unanswered questions and future research

The mechanisms for how teachers’ stereotypical perceptions of students might negatively affect students’ learning and performance have been hypothesised. These hypotheses require quantitative testing. Several studies, mainly with US college students, have measured stereotype threat and investigated the ways in which it can be manipulated experimentally.^13^ ^25^ Such studies could usefully be replicated with UK medical student populations. Measuring the relationships between stereotyping, teacher-student interactions, and student learning will be a challenge not least because of the socially unacceptable nature of ethnic stereotyping; however, psychologists have developed tools to measure implicit (subconscious) stereotyping^26^ and one recent study has found that white doctors’ implicit stereotypical views about white and black patients affected the diagnoses they made.^27^ Adapting these tools for use in medical education research could further aid understanding of the complex interactions between ethnicity, attitudes, behaviour, learning, and examination performance.

**What is already known on this topic**

Medical students from ethnic minorities under-perform academically compared with their white colleagues, for reasons that are unclear
US research has linked underperformance in college students from ethnic minorities to a psychological phenomenon known as stereotype threat—systematic differences in expectations and treatment owing to pervasive negative stereotypes

**What this study adds**

In one London medical school, Asian clinical medical students were negatively stereotyped by clinical teachers and students as overdependent on book learning and quiet in class
Participants believed these attributes explained underperformance in the Asian students, raising the possibility that stereotype threat may be occurring
Clinical teachers disliked teaching students they perceived as having these attributes, and students reported being unable to learn from unenthusiastic teachers, suggesting that negative stereotyping can adversely affect learning

## Notes

We thank Sara Belle for comoderating the focus groups, the participants for taking time to answer our questions, and those who made useful comments on earlier drafts of the paper and during oral presentations of the data. KW’s salary is supported by the General Medical Council. JC’s salary is supported by Cancer Research UK. The other authors are funded by the Higher Education Funding Council.

Ethical approval: Graduate School Ethics Committee and the NHS Research Ethics Committee.

Provenance and peer review: Not commissioned; externally peer reviewed.

## Notes

**Cite this as:** *BMJ* 2008;337:a1220
